# Report of a Rare Case of Beta-Thalassemia Major With Subperiosteal Hematomas

**DOI:** 10.7759/cureus.23770

**Published:** 2022-04-02

**Authors:** Parag S Mahajan, Jouhar J Kolleri, Sara Ait Souabni, Sakshi Prasad, El Habib Belhaddad, Hussain Mohammed

**Affiliations:** 1 Clinical Imaging Department, Hamad Medical Corporation, Doha, QAT; 2 Family Medicine, Faculty of Medicine and Pharmacy, Cadi Ayyad University, Marrakech, MAR; 3 Internal Medicine, National Pirogov Memorial Medical University, Vinnytsya, UKR; 4 Surgery Department, Hamad Medical Corporation, Doha, QAT

**Keywords:** magnetic resonance imaging, subperiosteal hematoma, musculoskeletal radiology, ultrasound, beta thalassemia major

## Abstract

Beta-thalassemia represents a range of hemoglobinopathies that are a consequence of an impairment in the synthesis of beta-globin chains. They result in different degrees of hemolysis and ineffective erythropoiesis, depending on the underlying mutations. They can lead to severe complications mainly resulting from anemia. However, there is no bleeding tendency in this disorder, and it is uncommon to see hematoma formation in affected patients.

To our knowledge, subperiosteal hematomas have been rarely described in the context of beta-thalassemia. Herein, we report a unique case of a 19-year-old boy who was diagnosed with transfusion-dependent beta-thalassemia and secondary hemochromatosis and developed atraumatic subperiosteal hematomas along the humerus.

## Introduction

Thalassemia represents a spectrum of hematological diseases where there is an impairment in the production of one or more globin chains of hemoglobin. This results in ineffective erythropoiesis and hemolysis, which can lead to severe anemia. Beta-thalassemia is the most prevalent type and is more frequently seen in the Mediterranean, the Middle East, and Southeast Asia. Nevertheless, because of immigration movements, their rates are increasing in Northern Europe and North America as well [1]. Beta-thalassemia can lead to serious complications, such as failure to thrive, bone deformity, organ damage, and susceptibility to infections [2]. However, bleeding complications are uncommon, and only a few case reports have been published describing bleeding localized in the brain, the iliopsoas muscle, and the spleen [2-6]. Herein, we report a case of a 19-year-old male diagnosed with beta-thalassemia major and secondary hemochromatosis, who presented with left shoulder pain and was later confirmed to have subperiosteal hematomas along the humerus.

## Case presentation

A 19-year-old male, a known case of beta-thalassemia receiving regular blood transfusion, with severe hepatic and cardiac iron overload on iron chelation, and delayed puberty on chronic gonadotrophin injections presented to the emergency department with a history of left shoulder pain for two weeks. The patient denied any history of trauma. He was vitally stable, and laboratory tests showed low levels of factor II, factor VIII, factor IX, and factor XI. On examination, there was local tenderness mainly in the shoulder anteriorly. There was redness and ecchymosis with restricted active range of shoulder movements. No neurological deficit was found. The capillary filling was positive with intact peripheral pulses.

**Figure 1:**
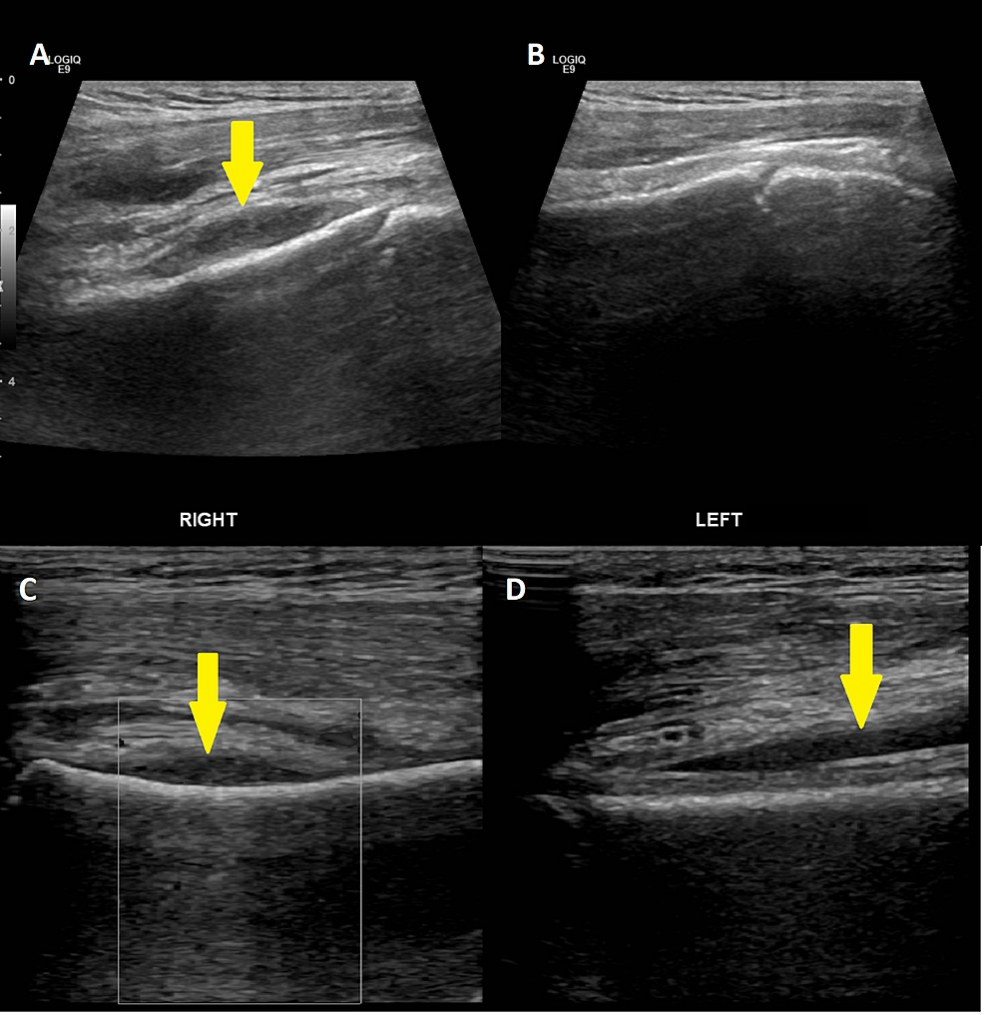
(A, C, and D) Ultrasound of the right shoulder showing a localized (subperiosteal) fluid collection adjacent to the humerus (yellow arrows) with elevation of the overlying muscles. (B) Left shoulder ultrasound showing normal morphology for comparison.

Radiographic evaluation of the right shoulder revealed no evident bony abnormality. Ultrasound Doppler of the right upper limb was performed to rule out deep vein thrombosis (DVT); however, there was no sonographic evidence of DVT in the right upper limb. Ultrasound of the right shoulder showed no evidence of joint effusion. There was a localized fluid collection measuring 2.1 x 0.5 cm adjacent to the humerus with elevation of the overlying muscles. Changes due to edema were also seen in the muscles adjacent to the humerus (Figure 1).

**Figure 2:**
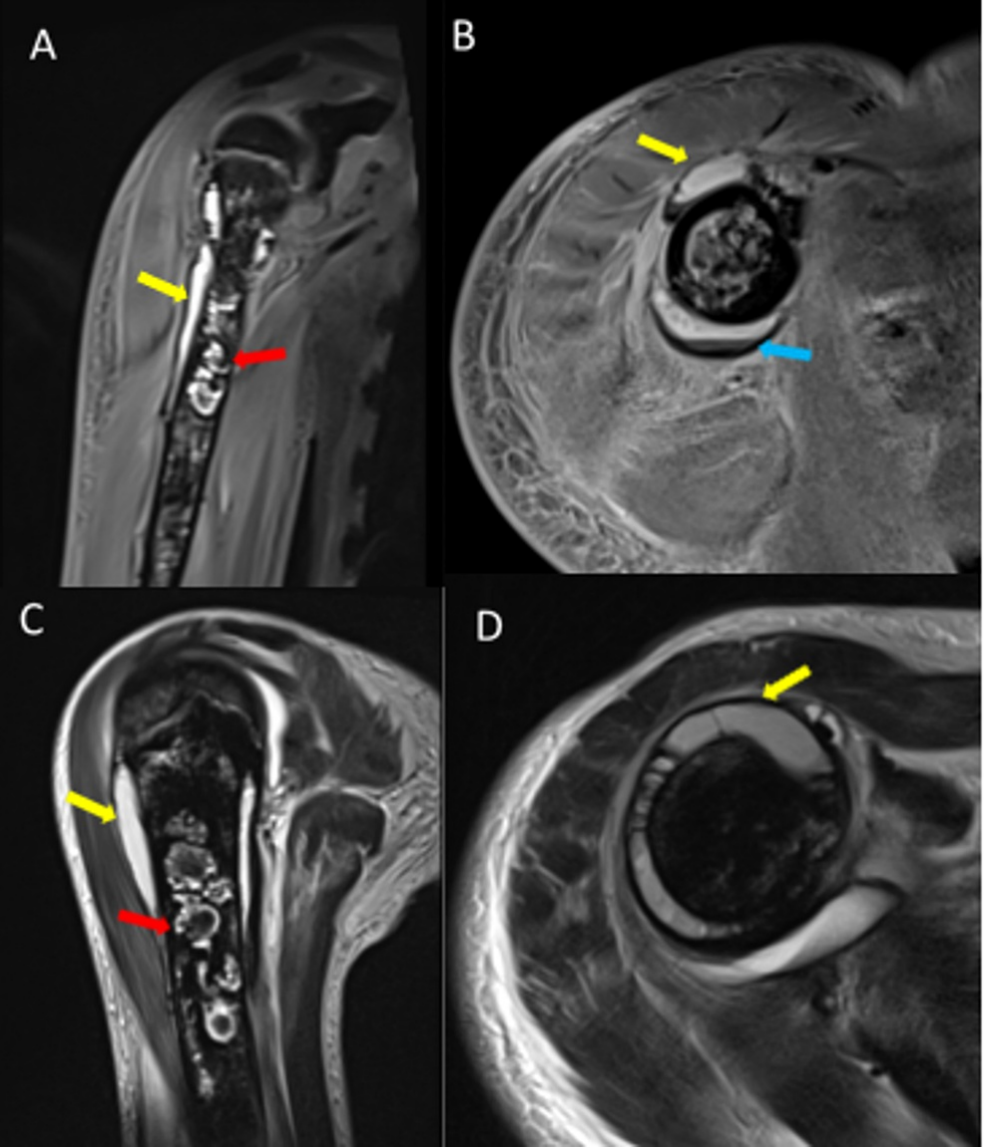
MRI of the right humerus. (A) Coronal T1, (B) axial T1 fat-suppressed, (C) sagittal T2, and (D) axial T2 images showing hyperintense localized subperiosteal fluid collections (yellow arrows) with fluid-debris levels (blue arrow). Ill-defined cystic changes in the proximal and distal metaphysis of the right humerus are likely due to iron deposition, chelation, and red marrow conversion (red arrows).

MRI of the right humerus with intravenous contrast was performed, which demonstrated heterogeneous marrow signal intensity involving the right humerus, which is the predominantly low intensity with ill-defined cystic changes in the proximal and the distal metaphysis. These changes were likely due to the iron deposition, chelation, and red marrow conversion. Localized complex subperiosteal fluid collections were noted circumferentially along the proximal right humerus; these measured around 70 mm in craniocaudal and 9 mm in transverse dimensions. Fluid debris levels were noted within a few of these collections. These were hyperintense on both T1- and T2-weighted images with hypointense debris, representing hematomas. Right shoulder and elbow joint effusions were evident. Diffuse edema/inflammatory changes of subcutaneous soft tissue and muscles of the right arm were noted with minimal fluid/blood collection in the fascial planes in the proximal part of the right arm, and most prominent fluid collection/hematoma in the fascial planes extending along the outer aspect of muscles in proximal and mid-third of the right arm measured approximately 121 mm in length and 6 mm in maximum thickness (Figure 2).

Ultrasound-guided aspiration biopsy was performed, which confirmed the presence of subperiosteal hematomas. The patient was treated with per-oral amoxicillin/clavulanate tablets, 1,000mg, twice daily for five days. The patient symptomatically improved and was discharged home with regular orthopedics follow-up.

## Discussion

Beta-thalassemia are hemoglobinopathies that originate from the modification of the beta-globin genes, leading to a decrease in their synthesis. To date, more than 300 alleles are known, among which only 40 account for more than 90% of all beta-thalassemia [7]. The severity of the disease is directly correlated with the level of decrease of the beta-globin, which results in wide phenotypic diversity, ranging from being asymptomatic (beta-thalassemia trait) to being dependent on transfusions (beta-thalassemia major) [1]. The symptoms of beta-thalassemia mainly result from the hemolysis of red blood cells; thus, they do not involve bleeding. However, six cases with bleeding complications in the context of beta-thalassemia have been reported, and different pathological mechanisms are suspected. A review of the literature was conducted, and the results are summarized in Table 1.

**Table 1:** Summary of case reports of beta-thalassemia complicated with hemorrhage ALT, alanine aminotransferase; AST, aspartate aminotransferase; CT, computed tomography; LDH, lactate dehydrogenase; MRI, magnetic resonance imaging; PT, prothrombin time; PTT, partial thromboplastin time

Case report	Year	Clinical findings	Imaging	Summary of laboratory results	Possible mechanism
Sanju et al. [2]	2019	A 7-year-old boy with beta-thalassemia major presented with severe anemia complicated with congestive heart failure. He was subsequently transfused over several days but developed seizures on day 9 of hospitalization.	CT scan revealed bilateral frontal hematoma with diffuse subarachnoid hemorrhage. Angiography showed spasmodic reaction to hemorrhage.	Coagulation profile and platelets: normal; ferritin levels: 2,497 ng/mL’ hepatic enzymes: AST/ALT of 105/99 U/L and LDH, of 3,040.9 U/L	Vessels fragility to blood viscosity after repeated and close transfusions in patients with chronic anemia
Dahal et al. [3]	2017	A 30-year-old man with a history of unclear sickling disorder was admitted for left-upper quadrant abdominal pain in the absence of trauma.	CT scan showed multiple splenic subcapsular lacerations and hematomas	Coagulation profile: normal; platelet count: 78,000/L; electrophoresis of hemoglobin: sickle cell beta-plus thalassemia	In the absence of a clear trigger to the splenic hematoma, this result might have been due to spontaneous bleed into chronic splenic infarcts facilitated by his hemoglobinopathy.
Padmakumari et al. [4]	2018	A 27-year-old man with transfusion-dependent beta-thalassemia under chelation therapy presented with acute back pain radiating to the groin, associated with swelling and tenderness of the right groin.	MRI showed extended right iliopsoas muscle hematoma with upper thigh expansion	Coagulation profile: aPTT of 41 sec, fibrinogen of 5.3 g/L, platelet count of 110,000/mm^3^, ferritin level of 3.247 ng/mL, hepatic enzymes were normal	Suspected mechanisms were multiple, ranging from tearing of muscle fibers, unrecognized minor trauma, secondary to hypersplenism, to coagulopathy due to liver injury by hemochromatosis
Svahn et al. [5]	2013	A 27-year-old woman with beta-thalassemia major presented with post-partum tonic-clonic seizures with no evident signs of preeclampsia.	MRI showed subarachnoid hemorrhage with no signs of chronic hypoperfusion; angiography showed complex vascular abnormalities such as intracranial carotid occlusion, carotid micro-aneurisms abnormally developed deep perforators and cortical arteries	Coagulation profile, hepatic enzymes, and ferritin levels: normal; platelet count: 800 giga/L; white blood cell count: 20 giga/L	Development of cortical collateral arterial network secondary to carotid occlusion by thromboembolic complications of thalassemia and hypercoagulable state
Lee [6]	1995	Case 1: A 12-year-old girl with beta-thalassemia major presented with severe headaches, loss of consciousness, and localized seizure.	CT scan showed massive intracerebral hemorrhage in the left occipito-parietal lobes and lateral ventricles	Coagulation profile: bleeding time of 3.5 min, clotting time of 5 minutes, PT of 19 sec, PTT of 130, platelet count of 40,000/mm^3^	Multifactorial mechanism: low platelet counts secondary to hypersplenism, and coagulopathy. Recent blood transfusion might have been a triggering factor for intracranial bleeding.
	1993	Case 2: A 7-year-old boy with beta-thalassemia major presented with acute headache, loss of consciousness, and seizures.	CT scan showed massive intracerebral hemorrhage in the left temporoparietal lobes and lateral ventricles	Coagulation profile: bleeding time of 4.5 min, PT of 17 sec, PTT of 58 sec, thrombin time of 7 sec, platelet count of 80,000/mm^3^	

The proposed mechanisms for the occurrence of bleeding complications in beta-thalassemia are multiple. Three cases of severe hemorrhage took place soon after repeated and relatively rapid blood transfusions [2,6]. In chronic anemia, blood vessels are used to low viscosity; hence, it is possible that they are unable to handle an abrupt increase in red blood cells (RBCs), which can be a trigger to hemorrhage [2]. Another possibility is an iron-induced liver injury causing severe coagulopathy [4]. The occurrence of bleeding secondary to micro-infarctions is a third eventuality [3,5]. The ischemia in the context of beta-thalassemia can be caused by a hypercoagulable state or by RBCs’ entrapment in sickle-cell beta plus thalassemia [3,5]. Another mechanism is the occurrence of severe thrombocytopenia secondary to hypersplenism [6].

Our case presented with a rare accumulation of blood in the subperiosteal region of the proximal right humerus. The diagnosis was made based on imaging and clinical features and confirmed by aspiration biopsy. Atraumatic pain in the right extremity with imaging of the region demonstrating circumferential, localized, and complex subperiosteal hematomas along the proximal humeral metaphysis has not been reported in the literature to the best of our knowledge. There have been a handful of published reports describing ossified subperiosteal hematomas in the calvarium, tibia, femur, iliac bone, and diaphysis of the humerus, but none that is non-ossified and related to beta-thalassemia [8-12]. Young age with a history of trauma, neurofibromatosis type 1 (NF1), and bleeding disorders such as hemophilia are risk factors implicated in the development of subperiosteal hematoma [12]. Vascular fragility and weakened periosteal attachment in NF1, pseudotumor in hemophilia, and weak attachment of the periosteum in non-traumatic cases of young adult, are some of the possible pathophysiological factors implicated in the development of this condition [9,13-15]. At the same time, traumatic injuries can also damage the nutrient vessels and cause periosteal avulsion injury leading to subperiosteal accumulation of blood [14]. The differential diagnoses in the patient could possibly include myositis ossificans and parosteal sarcomas. However, the absence of typical imaging features helped us exclude these pathologies. Ultrasound-guided aspiration biopsy confirmed subperiosteal hematomas.

Although ultrasound may be the first screening modality of choice to diagnose hematomas, additional investigations such as MRI or CT are necessary to make a final diagnosis, with MRI being superior in respect to diagnosing accompanying occult fractures in cases of traumatic hematomas. Transcatheter arterial embolization can be used in cases of expanding hematomas [16,17]. Surgical evacuation of the hematoma can be implicated in patients with accompanying neurological symptoms [18]. In our patient, further evaluation was not carried out since the hematoma did not expand on close follow-up using ultrasound. Conservative management with a good prognosis is described in the literature for all small, non-expanding hematomas and no accompanying neuropathy [18], with our patient falling in the same line of conservative management and further close orthopedic follow-up.

## Conclusions

Bleeding complications are uncommon in beta-thalassemia. Our patient is one of the rare described cases of atraumatic subperiosteal hematomas along the humerus in this context. The diagnosis was made by ultrasound and MRI and confirmed by aspiration biopsy, and the patient was treated conservatively.
